# Rapid Bolus Inflow into the Esophagus in a Patient with a Tracheostomy after Surgical Treatment for Dysphagia

**DOI:** 10.1007/s00455-025-10904-5

**Published:** 2025-11-16

**Authors:** Kenjiro Kunieda, Takashi Shigematsu, Hideaki Kanazawa, Akiko Nomoto, Kyoko Hojo, Ichiro Fujishima

**Affiliations:** 1 Department of Neurology, Gifu University Graduate School of Medicine, Gifu, 1-1 Yanagido, 501-1194 Japan; 2 Department of Rehabilitation Medicine, Hamamatsu City Rehabilitation Hospital, Hamamatsu, Shizuoka Japan; 3 Swallowish Clinic, Chuo-ku, Tokyo, Japan; 4 Department of Dentistry, Hamamatsu City Rehabilitation Hospital, Hamamatsu, Shizuoka Japan; 5 Department of Rehabilitation, Hamamatsu City Rehabilitation Hospital, Hamamatsu, Shizuoka Japan

**Keywords:** High-resolution manometry, Negative pressure, Brain tumor, Rehabilitation, Lower esophageal sphincter, Inspiratory

## Abstract

This report describes a case in which a patient with an open tracheostomy, following surgery for severe dysphagia, acquired vacuum swallowing and exhibited rapid bolus inflow into the esophagus. A 39-year-old man with bulbar palsy caused by medullary surgery demonstrated impaired pharyngeal contraction and upper esophageal sphincter opening. After undergoing laryngeal suspension and cricopharyngeal myotomy, videofluoroscopic evaluation of swallowing revealed rapid passage of the bolus from the pharynx into the esophagus. High-resolution manometry demonstrated markedly negative intraesophageal pressure accompanied by simultaneous elevation of lower esophageal sphincter pressure during swallowing. These findings suggest that the patient had spontaneously acquired vacuum swallowing despite the presence of a tracheostoma communicating with the atmosphere. Recognition of this compensatory mechanism is important because it may facilitate bolus transport in individuals with tracheostomy. Increased awareness of this swallowing pattern may prevent underdiagnosis and offer new insights into rehabilitation strategies for dysphagia.

**Supplementary Information:**

The online version contains supplementary material available at 10.1007/s00455-025-10904-5.

## Case Presentation

**Fig. 1:**
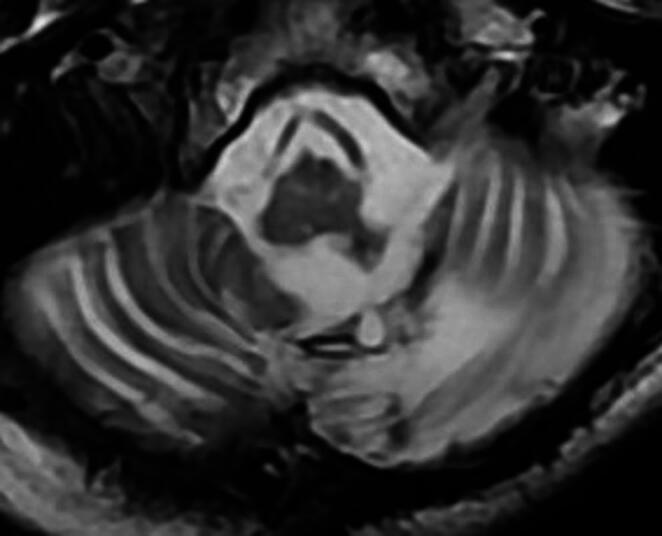
T2-weighted image showing high signal intensity in the left medullary and cerebellum

A 39-year-old male diagnosed with von Hippel-Lindau disease underwent multiple medullary and cerebellar hemangioblastoma resections. He underwent the first resection at 27 years old; however, the tumor recurred at the same site, necessitating a second surgery at 36 years old. After surgery, the patient developed severe dysphagia caused by bulbar palsy due to medullary damage (Fig. 1). The patient also developed left-sided limb and truncal ataxia. Postoperatively, the patient underwent salivary aspiration, and a tracheostomy was performed 1 month after the surgery. Severe dysphagia was classified as level 2 via the Food Intake LEVEL Scale (FILS) (swallowing training not using food was performed) [1]. Nutritional support was provided via enteral feeding. Swallowing rehabilitation, including balloon dilation therapy (BDT) for the impaired upper esophageal sphincter (UES), was continued [2–4]; however, he developed aspiration pneumonia.

**Fig. 2:**
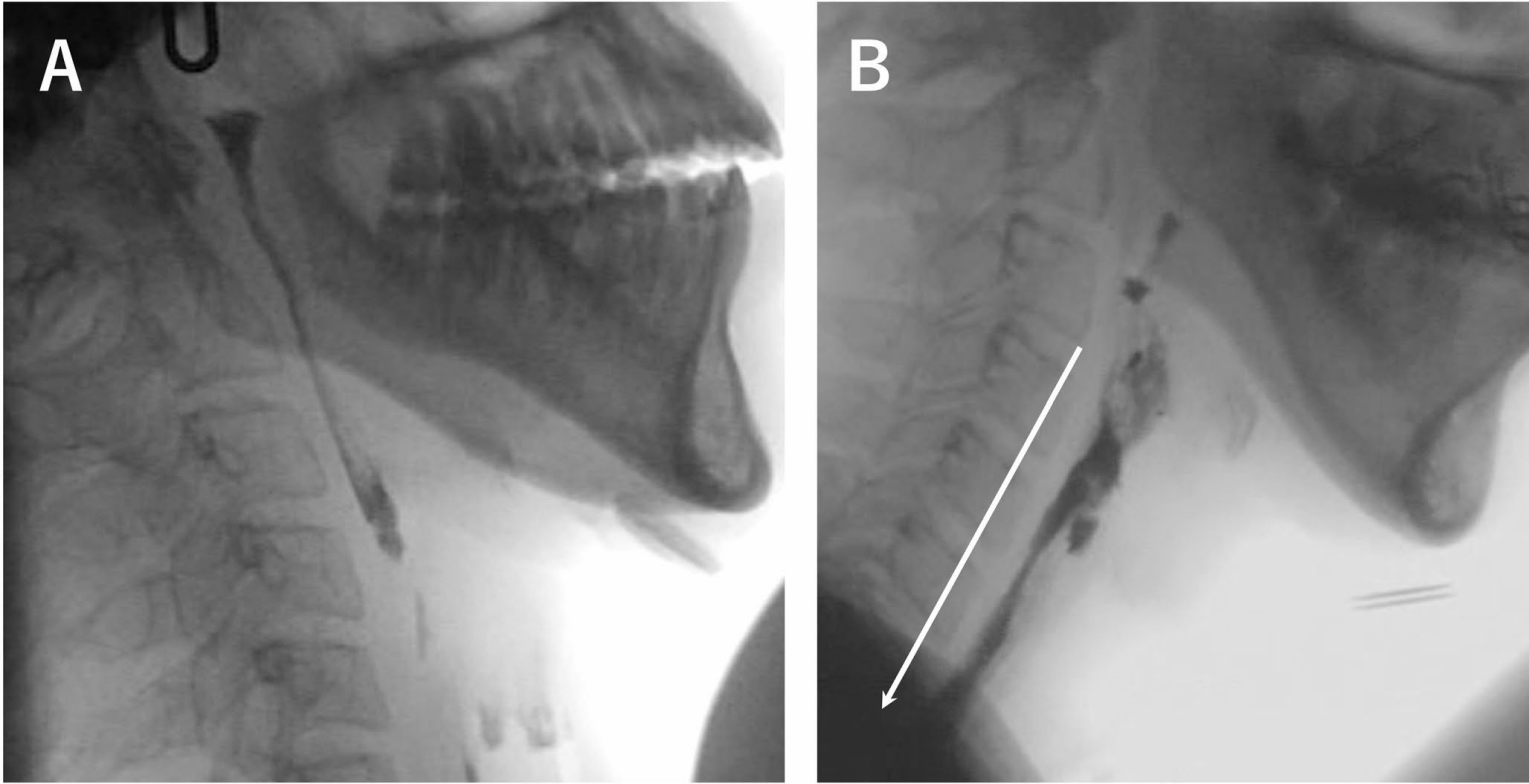
Videofluoroscopic Analysis of Bolus Movement Before and After Surgery. (**A**) Preoperative: Bolus residue in the pyriform sinus regurgitated into the nasopharynx during swallowing. The bolus did not enter the esophagus. (**B**) Postoperative: The bolus rapidly flowed into the esophagus during swallowing, as if being sucked in

Five months after surgery, the patient was transferred to our rehabilitation hospital for intensive swallowing therapy to treat severe dysphagia. The patient continued swallowing rehabilitation; however, a videofluoroscopic examination of swallowing (VF) revealed that the bolus did not completely pass through the UES (Fig. 2). Therefore, surgical interventions were performed to improve swallowing function, including cricopharyngeal myotomy, laryngeal suspension, and epiglottoplasty. One month after surgery, the laryngeal edema improved. VF showed that the bolus had passed through the pharynx, and BDT was restarted. The patient was able to resume oral intake. Dysphagia improved to FILS level 8 (the patient ate three meals by excluding food that was particularly difficult to swallow). The tracheal cannula was removed, leaving the stoma open to the atmosphere. Ultimately, he was able to consume a normal oral diet. Subsequently, swallowing function improved to FILS level 9 (there was no dietary restriction, and the patient ingested three meals orally; however, medical matters were considered). Six months after surgery, the patient was discharged from the hospital and could continuously consume food orally. As part of the follow-up care, VF and high-resolution manometry (HRM) were performed 2 years after surgery for dysphagia to evaluate swallowing function.

VF revealed that the bolus flowed rapidly from the pharynx into the esophagus (Fig. 2 and Video 1). Although some residue remained in the pharynx after swallowing, it subsequently flowed into the esophagus. Sliced jelly and thickened liquid were used in this study. No aspirations or significant pharyngeal residues were observed.

## What were the Underlying Physiological Mechanisms of This Swallowing Method?

Manometric data were obtained using a solid-state manometry catheter assembly (outer diameter: 4.2 mm) with 36 circumferential pressure sensors placed 1 cm apart. The catheter was inserted intranasally and positioned to obtain data from the hypopharynx to the stomach beyond the high-pressure zone of the lower esophageal sphincter (LES). The minimum esophageal pressure (esophageal Pmin) and maximum LES pressure (LES Pmax) were recorded (Table 1). Eight swallowing events were evaluated during data collection, and all exhibited negative pressure in the esophagus and increased LES pressure. Additionally, minimum pressures during inspiration and expiration were measured and recorded.

**Table 1:** Esophageal and LES pressure

	Vacuum swallowing	Expiration	Inspiration
Pmin (mmHg)	–25.5 ± 7.7	–4.2	–10.2
Pmax (mmHg)	77.0 ± 14.9	15.0	27.8

Pmin, intraesophageal minimum pressure; Pmax, max pressure of the lower esophageal sphincter (LES)

**Fig. 3:**
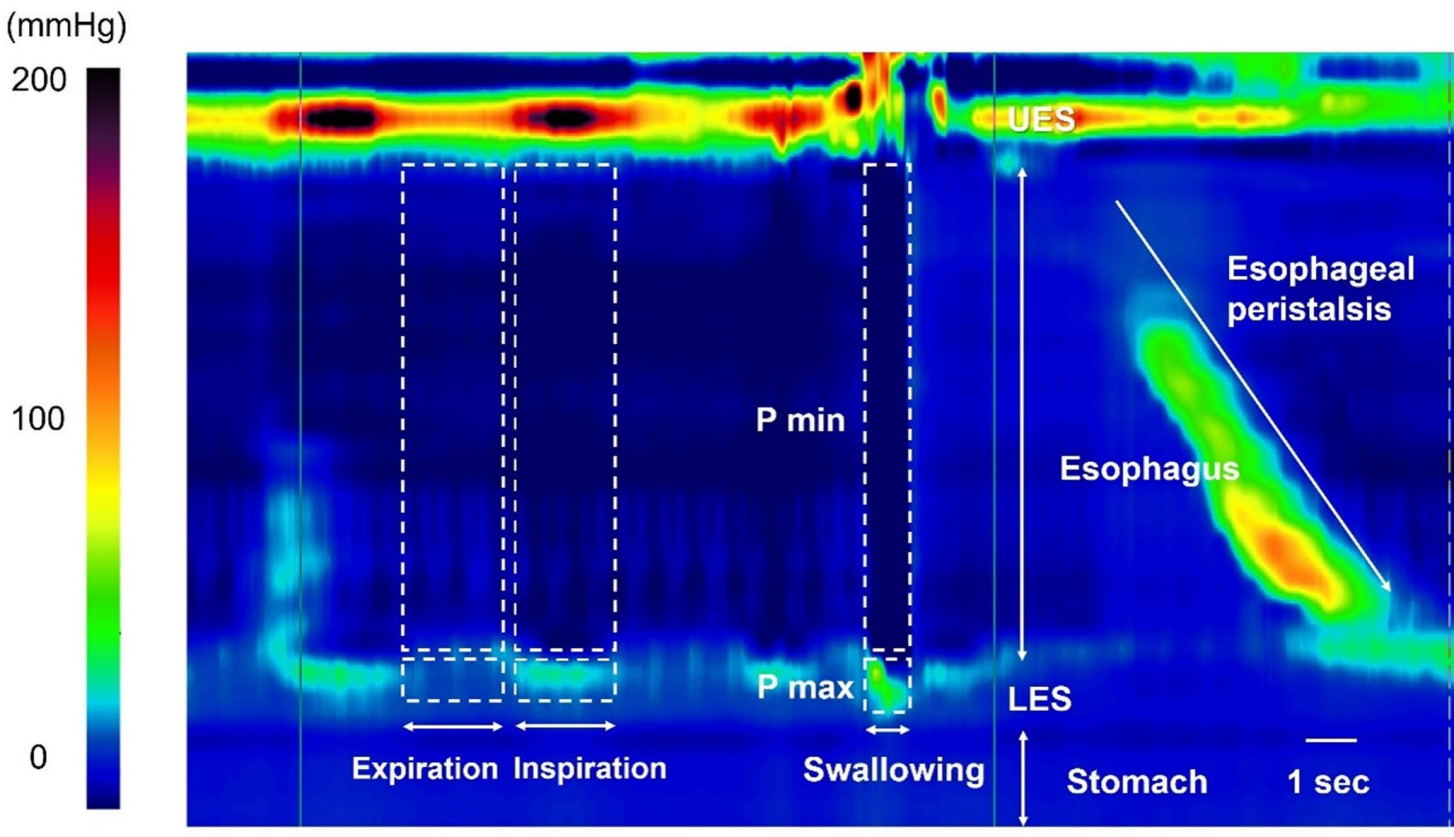
The topography of vacuum swallowing on high-resolution manometry. Negative pressure is observed in the esophagus during vacuum swallowing (Pmin). The LES muscle constriction was measured (Pmax) and was within a comparable range during inspiration and expiration. UES, upper esophageal sphincter; LES, lower esophageal sphincter

Vacuum swallowing is a unique swallowing maneuver that facilitates bolus passage from the pharynx into the esophagus by generating negative pressure in the esophagus [1, 5–10] (Fig. 3). This maneuver has primarily been observed in patients with dysphagia caused by lateral medullary syndrome (LMS). It is a well-suited adaptation to address the pathophysiology of reduced pharyngeal contractility and impaired UES opening. VF demonstrated that a bolus is sucked from the pharynx into the esophagus during vacuum swallowing, even in the absence of significant pharyngeal contraction [5]. Furthermore, this maneuver is adaptable for clearing pharyngeal residues in the pyriform sinus [8]. Interestingly, healthy individuals are capable of reproducing vacuum swallowing [9, 10], and both healthy individuals and patients can learn this swallowing maneuver through instruction [8–10].

The mechanism underlying vacuum swallowing involves strong contraction of the inspiratory muscles during swallowing. HRM revealed a specific pattern of esophageal pressure characteristics, including strong negative intraesophageal pressure and contraction of the LES [5, 7–10]. The increase in LES pressure was attributed to contraction of the diaphragm, a major inspiratory muscle. Furthermore, the prominence of the sternocleidomastoid muscle and clavicle during vacuum swallowing reflects the negative pressure in the thoracic cavity [5, 9, 10].

To generate a strong negative pressure in the thoracic cavity during swallowing, closure of the glottis is required to prevent airflow into the airway [5, 9, 10]. However, it is unclear whether patients with dysphagia with an open tracheostoma communicating with the atmosphere can perform vacuum swallowing. This creates negative pressure in the thoracic cavity during vacuum swallowing.

## Discussion

This case is unique because a patient with dysphagia independently acquired vacuum swallowing despite the presence of an open tracheostomy. VF demonstrated that the bolus was rapidly sucked from the pharyngeal cavity into the esophagus. HRM revealed that negative pressure was created in the esophagus during swallowing, accompanied by increased LES pressure, reflecting constriction of the diaphragm. These findings are consistent with previous reports [5, 7–10].

The most important finding was that a strong negative pressure was generated in the thoracic cavity, even when the lower respiratory tract was open to the atmosphere through the tracheostoma. When inspiratory effort is performed with airway obstruction caused by a closed glottis, a contracted pharynx, and tongue attachment to the palate during vacuum swallowing, a strong negative pressure is created in the thoracic cavity [5, 9, 10]. In this patient, the transient negative intrathoracic pressure during vacuum swallowing was likely generated by a discrepancy between the rapid expansion of the thorax and the delayed expansion of the lungs, reflecting differences in compliance between the chest wall and the lungs. Similar to some previously reported cases, this patient acquired the ability to perform vacuum swallowing spontaneously, without any specific instruction. However, compared to previous reports, this patient exhibited a relatively weaker negative pressure in the esophagus during vacuum swallowing. This may be because the airway was open to the atmosphere through the tracheostomy.

In this case, the swallowing disorder was presumed to be bulbar-type dysphagia caused by a brainstem lesion. The patient exhibited weak pharyngeal constriction and an impaired UES opening, for which laryngeal elevation surgery and cricopharyngeal myotomy were performed. In addition to the surgical interventions, the patient’s self-acquisition of vacuum swallowing may have contributed to the improvement in pharyngeal bolus passage.

Notably, even patients with dysphagia who undergo tracheostomy can achieve vacuum swallowing with appropriate instructions, as reported in previous studies [9, 10]. It is generally considered difficult to generate sufficient negative intrathoracic pressure during swallowing in patients with tracheostomy, because inspiratory effort causes air to flow into the airway, preventing negative intrathoracic pressure generation. However, in this case, effective negative intrathoracic pressure was generated, resulting in vacuum swallowing. This finding is particularly unique even in the presence of an open tracheostomy. Although the patient underwent an open tracheostomy, a stronger negative pressure in the esophagus might have been generated when a valve was used with the tracheal cannula.

At present, vacuum swallowing can only be clearly identified by manometric study. When VF shows very rapid pharyngeal bolus passage, as in this case, performing manometry may reveal this phenomenon. Therefore, some cases may not have been recognized simply because pressure dynamics were not evaluated. Vacuum swallowing may be acquired as a compensatory swallowing method in patients with dysphagia due to weak pharyngeal contraction and impaired UES opening. We believe that vacuum swallowing may also occur in other neurological dysphagia disorders, but it has not been reported to date due to the difficulty of diagnosing it and the fact that many clinicians are not yet aware of this swallowing phenomenon. Further studies are needed to determine whether this phenomenon can also be observed in other similar conditions.

In summary, we report a unique case in which a patient with dysphagia and an open tracheostomy independently acquired vacuum swallowing. Further research is needed to determine whether patients undergoing tracheostomy can consistently acquire vacuum swallowing following instructions.

## Supplementary Information

Below is the link to the electronic supplementary material.

Supplementary Material 1
